# Isatuximab plus pomalidomide and dexamethasone in frail individuals with relapsed/refractory multiple myeloma in Japan

**DOI:** 10.1007/s12185-024-03904-y

**Published:** 2024-12-27

**Authors:** Nami Tagami, Michihiro Uchiyama, Kenshi Suzuki, Heigoroh Shirai, Takeshi Seto, Shinsuke Iida

**Affiliations:** 1 Oncology Medical in Specialty Care, Sanofi K.K., Tokyo, Japan; 2 Department of Hematology, Japanese Red Cross Society Suwa Hospital, Suwa, Japan; 3 Myeloma/Amyloidosis Center, Japanese Red Cross Medical Center, Tokyo, Japan; 4 Medical Affairs, Post-Authorization Regulatory Studies, Sanofi K.K., Tokyo, Japan; 5 Department of Hematology and Oncology, Nagoya City University Institute of Medical and Pharmaceutical Sciences, Kawasaki 1, Mizuno-cho, Mizuno-ku, Nagoya, Aichi 467-8601 Japan; 6 Present Address: Department of Diabetes, Endocrinology and Hematology, Dokkyo Medical University Saitama Medical Center, Saitama, Japan

**Keywords:** Frailty, Isatuximab, Japan, Real-world, Relapsed or refractory multiple myeloma

## Abstract

This post-marketing surveillance (PMS) assessed the safety and effectiveness of isatuximab plus pomalidomide and dexamethasone (Isa-Pd) for relapsed or refractory multiple myeloma (RRMM) in frail individuals during real-world use in Japan. Data from all individuals with RRMM treated with Isa-Pd in Japan between October 2020 and October 2021 were collected, with follow-up continued up to 12 months after starting Isa-Pd or until discontinuation. In the overall PMS population, 40 participants were classified as frail (33.3%) and 29 as fit/intermediate (24.2%), and 51 had no frailty score (42.5%). Incidence of adverse drug reactions in each group was 77.5%, 65.5%, and 37.3%. In frail versus fit/intermediate participants, bone-marrow suppression occurred in 72.5% versus 44.8%, infectious diseases in 17.5% versus 10.3%, and infusion-related reactions in 7.5% versus 3.5%. Heart failure occurred in one participant with no frailty score. The rates of overall response and very good partial response or better were higher (*p* = 0.101) in fit/intermediate participants (56.0% and 36.0%) than in frail participants (38.5% and 18.0%). Rates of treatment discontinuation due to disease progression were similar between groups. These findings support the safety and effectiveness of Isa-Pd for frail individuals with RRMM in real-life settings in Japan.

**Supplementary Information:**

The online version contains supplementary material available at 10.1007/s12185-024-03904-y.

## Introduction

With a median age at diagnosis of 69 years, multiple myeloma (MM) is predominantly a disease of the elderly [1]. Because of a greater risk of frailty (i.e., decline in physiological function, dependency, vulnerability to stressors, and greater frequency of comorbidities), current treatments are less effective and not as well tolerated in older and/or less fit individuals with MM, resulting in increased morbidity and mortality [2].

Following the introduction of the International Myeloma Working Group (IMWG) frailty score based on age, comorbidities, and functional status, and designed to guide therapy selection in individuals with newly diagnosed MM [3], other simplified frailty scores have been developed [4, 5]. Analysis of data from the registrational ICARIA-MM study, using a frailty score based on baseline characteristics (i.e., age, modified Charlson Comorbidity Index [mCCI], and Eastern Cooperative Oncology Group performance status [ECOG PS]), showed that clinical response, long-term treatment benefit, and the safety profile of isatuximab plus pomalidomide and dexamethasone (Isa-Pd) in frail individuals were consistent with those in the overall study population, confirming the feasibility of this triplet regimen in the frail population [5].

Due to the small number of Japanese individuals (*n* = 13) included in the ICARIA-MM study [6, 7], the Japanese Pharmaceuticals and Medical Devices Agency (PMDA) requested an all-case post-marketing assessment of isatuximab as a condition of approval in Japan [8, 9]. Thus, post-marketing surveillance (PMS) was initiated to collect data on the use of isatuximab in Japan, with the aim of investigating the real-world safety and effectiveness of Isa-Pd in individuals with relapsed or refractory MM (RRMM) [9]. Full results of the PMS have been reported and demonstrated the safety and effectiveness of Isa-Pd in real-life clinical settings in Japan, with no new safety signals identified, indicating that no additional safety measures are required in Japanese individuals [9]. The current subgroup analysis investigated the impact of frailty on safety and clinical outcomes in Japanese participants in the PMS treated with Isa-Pd.

## Materials and methods

### Study design

The PMS design has been described previously [9], but briefly, it was a multicenter, uncontrolled, non-comparative, observational study. All participants in the PMS had received isatuximab for RRMM at Japanese medical institutions and provided written informed consent [9]. Participants were followed for up to 12 months after the start of isatuximab administration or until treatment discontinuation. The current subgroup analysis utilized the simplified frailty score previously used in the ICARIA-MM study [4, 5] to investigate the impact of frailty on safety and clinical outcomes in Japanese participants in the PMS treated with Isa-Pd. The study was performed in compliance with the guidelines for Good Post-marketing Study Practice (GPSP) in Japan and the Declaration of Helsinki.

### Study population and treatment

All individuals treated with isatuximab for RRMM at Japanese medical institutions from October 31, 2020, to October 31, 2021, were registered. Isatuximab 10 mg/kg was administered by intravenous infusion (in combination with pomalidomide and dexamethasone). The 28-day treatment cycles consisted of four infusions (on Days 1, 8, 15, and 22) in the first cycle and two infusions (on Days 1 and 15) in subsequent cycles.

### Outcome measures

The PMS evaluated safety (i.e., adverse drug reactions [ADRs] classified using the systems order class and preferred terms [PTs] from the Medical Dictionary for Regulatory Activities, Japanese version [MedDRA-J]), ADRs of special interest (i.e., infusion-related reactions, bone-marrow suppression, infections, and cardiac disorders), and effectiveness of Isa-Pd (evaluated at the end of the final cycle of treatment using IMWG response criteria [9, 10]).

Using a previously described algorithm [5, 11], participants were assigned to frailty categories based on the sum of scores for age (0 if < 75 years, 1 if 75–80 years, and 2 if > 80 years), mCCI (0 if the CCI ≤ 1 and 1 if the CCI > 1), and ECOG PS (0 if the ECOG PS was 0, 1 if the ECOG PS was 1, and 2 if the ECOG PS was 2). The mCCI was calculated using detailed medical history recorded during a screening visit and coded using the MedDRA-J and PTs for medical history matching those of the CCI [11]. Participants without a medical history were considered to have a missing frailty score. Total frailty scores of 0, 1, or ≥ 2 were classified as fit, intermediate, or frail, respectively. To simplify comparisons between frail and non-frail participants, the fit and intermediate groups were combined for analyses.

### Statistical analyses

Data were analyzed using descriptive statistics, with categorical variables presented as the number and proportion of participants and continuous variables presented as means ± standard deviation (SD) and medians (ranges). Comparisons between frailty subgroups were purely descriptive, with the exception of the difference in overall response rate (ORR) between groups, where *p*-values were calculated using the Fisher’s exact test and the Cochran–Armitage test, with a two-side significance level set at 5%. Statistical analyses were conducted using SAS^®^ software (Cary, NC, USA), version 9.4 or later.

## Results

Between October 20, 2020 and April 19, 2022, a total of 120 participants with RRMM were treated with Isa-Pd and were included in the PMS [9]. The mean ± SD age of participants was 70.2 ± 9.2 years, and most participants (74.2%) were aged ≥ 65 years [9].

The overall PMS population comprised 40 (33.3%) frail participants, 29 (24.2%) fit/intermediate participants, and 51 (42.5%) participants with a missing frailty score (Table 1). The median (range) age of the frail participants and fit/intermediate participants was 75 (62, 90) and 67 (51, 79) years, respectively. All participants were receiving pretreatment medications for RRMM at baseline and 47.5% and 37.9% of participants in both the frail and fit/intermediate groups had Revised International Staging System (R-ISS) Stage II disease at the start of the PMS. All participants in the fit/intermediate group had an ECOG PS of 0 or 1, while 30.0% and 25.0% of frail participants had an ECOG PS of 2 or 3, respectively. Fewer frail participants completed the ≥ 12-month observation period than fit/intermediate participants or those with missing frailty scores (17.5% vs 31.0% and 31.4%, respectively; Table 2). While all participants received 10.0 mg/kg of isatuximab per dose, the doses of pomalidomide and dexamethasone were numerically lower in the frail subgroup versus the fit/intermediate subgroup (Table 3). Participants in the frail subgroup were also treated with isatuximab for a shorter period of time (72.0 days) than participants in the fit/intermediate subgroup (97.0 days; Table 3).

**Table 1:** Baseline patient characteristics

Variable	Frail (*n* = 40)	Fit/intermediate (*n* = 29)	Missing frailty score (*n* = 51)
*Sex, n (%)*			
Male	27 (67.5)	16 (55.2)	28 (54.9)
Female	13 (32.5)	13 (44.8)	23 (45.1)
*Age, years, median (range)*	75.0 (62, 90)	67.0 (51, 79)	70.0 (46, 86)
*Age category, n (%)*			
< 65 years	5 (12.5)	10 (34.4)	16 (31.4)
≥ 65 years to < 75 years	13 (32.5)	16 (55.2)	22 (43.1)
≥ 75 years	22 (55.0)	3 (10.3)	13 (25.5)
*Pretreatment medications, n (%)*	40 (100.0)	29 (100.0)	51 (100.0)
*ISS stage, n (%)*			
Stage I	6 (15.0)	6 (20.7)	4 (7.8)
Stage II	17 (42.5)	10 (34.5)	17 (33.3)
Stage III	17 (42.5)	12 (41.4)	14 (27.5)
Unknown	0	1 (3.4)	16 (31.4)
*R-ISS stage, n (%)*			
Stage I	3 (7.5)	5 (17.2)	2 (3.9)
Stage II	19 (47.5)	11 (37.9)	17 (33.3)
Stage III	12 (30.0)	8 (27.6)	10 (19.6)
Unknown	6 (15.0)	5 (17.2)	22 (43.1)
*ECOG PS*			
0	0	12 (41.4)	13 (25.5)
1	18 (45.0)	17 (58.6)	16 (31.4)
2	12 (30.0)	0	18 (35.3)
3	10 (25.0)	0	2 (3.9)
4	0	0	1 (2.0)
Unknown	0	0	1 (2.0)
*Modified CCI score*			
≤ 1	36 (52.2)	44 (89.8)	83 (100.0)
> 1	33 (47.8)	5 (10.2)	0

ECOG PS, Eastern Cooperative Oncology Group performance status; ISS, International Staging System; R-ISS, Revised International Staging System

**Table 2:** Participant disposition according to frailty score

Parameter	Frail (*n* = 40)	Fit/intermediate (*n* = 29)	Missing frailty score (*n* = 51)
*Completed observation period of* ≥ *12 months, n (%)*	7 (17.5)	9 (31.0)	16 (31.4)
*Reasons for discontinuation*			
Progressive disease	15 (37.5)	11 (37.9)	21 (41.2)
Death	7 (17.5)	3 (10.3)	1 (2.0)
Primary disease	6 (15.0)	3 (10.3)	1 (2.0)
Other than primary disease	1 (2.5)	0	0
Adverse events	4 (10.0)	2 (6.9)	4 (7.8)
Participant withdrawal	2 (5.0)	0	1 (2.0)
Lost to follow-up	0	4 (13.8)	5 (9.8)
Other	5 (12.5)	0	3 (5.9)

**Table 3:** Treatment duration and relative dose intensity according to frailty score

Parameter	Frail (*n* = 40)	Fit/intermediate (*n* = 29)	Missing frailty score (*n* = 51)
*Therapeutic agents for RRMM*			
Isatuximab			
Average single dose, mg/day, median (range)	10.0 (9.0, 10.0)	10.0 (10.0, 10.0)	10.0 (8.5, 10.0)
Treatment duration^a^, days median (range)	72.0 (1.0, 228.0)	97.0 (8.0, 220.0)	82.0 (1.0, 223.0)
Pomalidomide			
Average single dose, mg/day, median (range)	2.35 (1.0, 4.0)	3.07 (1.1, 4.0)	4.0 (1.0, 4)
Treatment duration^a^, days median (range)	83.0 (1.0, 316.0)	110.0 (8.0, 294.0)	98.5 (1.0, 301.0)
Dexamethasone			
Average single dose, mg/day, median (range)	20.0 (4.0, 40.0)	29.4 (8.0, 40.0)	20.0 (3.3, 40.0)
Treatment duration^a^, days median (range)	74.0 (1.0, 294.0)	93.0 (8.0, 730.0)	88.0 (1.0, 323.0)

RRMM, relapsed or refractory myeloma

^a^Excludes rest days

### Safety outcomes

Overall, 77.5% of frail participants experienced ADRs (Table 4). Corresponding percentages in the fit/intermediate and missing frailty score groups were 65.5% and 37.3%. The most common any-grade non-hematologic ADR in frail participants was infusion-related reaction (7.5% vs 3.5% in fit/intermediate participants), and in fit/intermediate participants was hypotension (6.9% vs 0% in frail participants), hypoxia (6.9% vs 0%), and oropharyngeal discomfort (6.9% vs 0%). In participants with a missing frailty score, the most common non-hematologic ADR was infusion-related reactions (7.8%).

**Table 4:** Most common adverse drug reactions occurring in ≥ 1 participant in any subgroup according to frailty score in individuals treated with isatuximab plus pomalidomide and dexamethasone

*n* (%)	Frail (*n* = 40)	Fit/intermediate (*n* = 29)	Missing frailty score (*n* = 51)
*Any-grade ADR*	31 (77.5)	19 (65.5)	19 (37.3)
Neutrophil count decreased	16 (40.0)	6 (20.7)	9 (17.7)
Neutropenia	7 (17.5)	1 (3.5)	1 (2.0)
Anemia	6 (15.0)	4 (13.8)	2 (3.9)
Platelet count decreased	6 (15.0)	4 (13.8)	5 (9.8)
White blood cell count decreased	4 (10.0)	4 (13.8)	1 (2.0)
Infusion-related reaction	3 (7.5)	1 (3.5)	4 (7.8)
Febrile neutropenia	2 (5.0)	3 (10.3)	1 (2.0)
Fever	2 (5.0)	0	0
Pneumonia	1 (2.5)	1 (3.5)	2 (3.9)
Hypertension	0	0	2 (3.9)
Hypotension	0	2 (6.9)	0
Hypoxia	0	2 (6.9)	0
Oropharyngeal discomfort	0	2 (6.9)	1 (2.0)

ADRs were coded according to the systems order class and preferred terms of the MedDRA-J

ADR, adverse drugs reaction; MedDRA-J, Medical Dictionary for Regulatory Activities, Japanese version

Bone-marrow suppression including neutropenia and decreased neutrophil count was more common in frail participants than in fit/intermediate participants or those with a missing frailty score (72.5% vs 44.8% and 27.5%, respectively). When the PTs for neutropenia (‘Febrile neutropenia’, ‘Neutropenia’, ‘Neutrophil count decreased’) were grouped together, the incidence was 62.5% (25/40) in the frail group, 34.5% (10/29) in the fit/intermediate group, and 21.6% (11/51) in the group with missing frailty scores. This was mostly driven by increased rates of decreased neutrophil count and neutropenia in frail participants (Table 4). However, rates of anemia, decreased platelet count, and decreased white blood cell counts were similar in frail and fit/intermediate participants. Also, infectious diseases were slightly more common in frail participants than in fit/intermediate participants or those with a missing frailty score (17.5% vs 10.3% and 7.8%, respectively).

Only one heart disorder ADR was reported during the study period, which was a heart failure event in one participant with a missing frailty score. Death during treatment with Isa-Pd occurred in 17.5% of frail participants, 10.3% of fit/intermediate participants, and 2.0% of participants with a missing frailty score (Table 2). In total, death occurred in 9 patients due to adverse events ([AEs]; Supplemental Table 1).

### Effectiveness outcomes

**Fig. 1:**
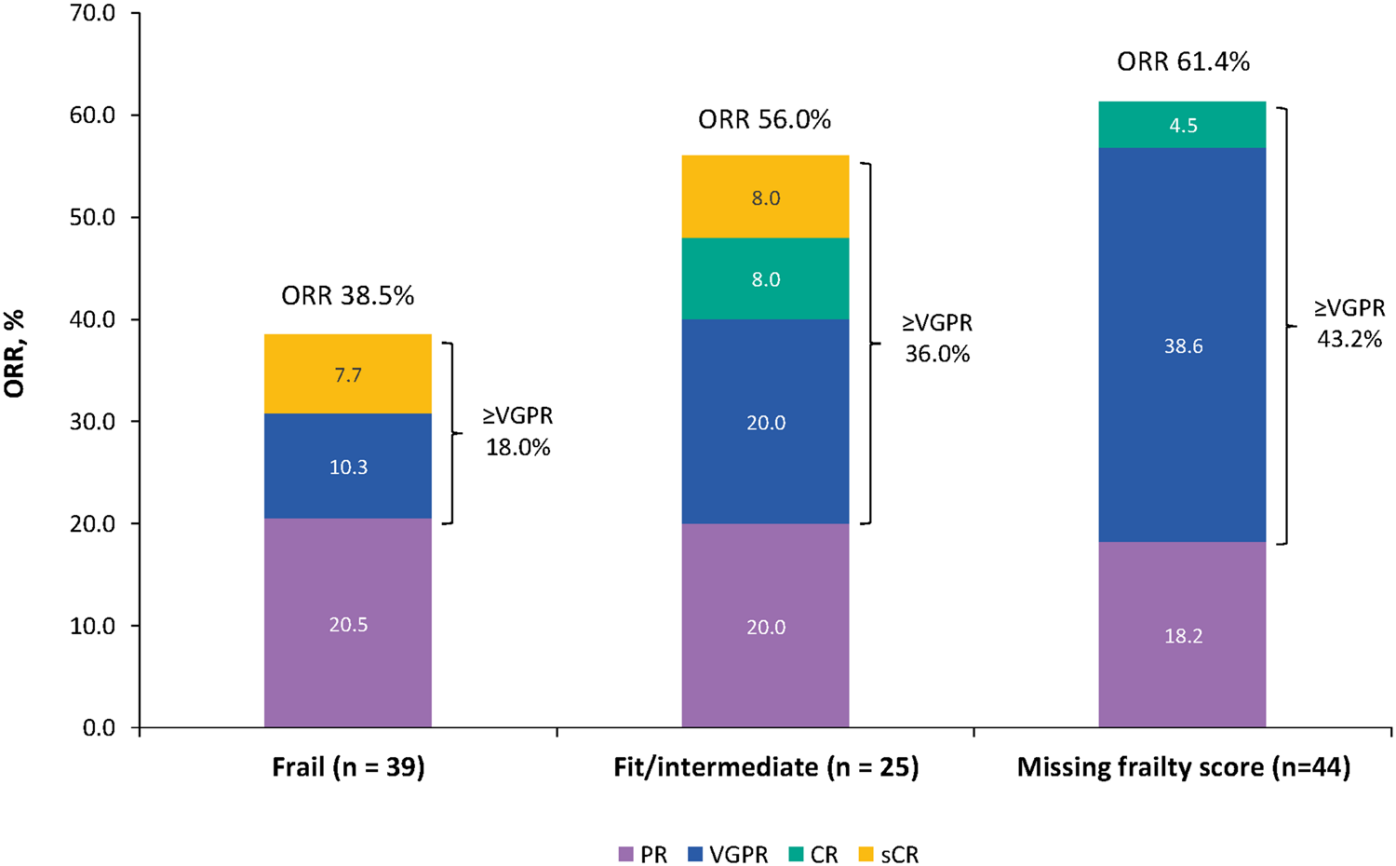
Overall response rate in frail versus fit/intermediate participants treated with isatuximab plus pomalidomide and dexamethasone. Response was assessed at the end of the final treatment cycle using International Myeloma Working Group criteria [10]. CR, complete response; ORR, overall response rate; PR, partial response; sCR, stringent complete response; VGPR, very good partial response

While the ORR and very good partial response or better (≥ VGPR) rate were higher in fit/intermediate participants (56.0% and 36.0%, respectively) than in frail participants (38.5% and 18.0%), the differences between the two groups were not statistically significant (*p* = 0.101; Fig. 1). Similar proportions of frail, fit/intermediate, and participants with a missing frailty score had stable disease (23.1%, 24.0%, and 13.6%, respectively). While more frail participants than fit/intermediate participants and those with missing frailty scores had progressive disease (33.3% vs 20.0% and 22.7%, respectively), discontinuations due to disease progression were similar between the groups (37.5% vs 37.9% and 41.2%; Table 2). There were no cases where the status of the investigation was unknown during the study.

## Discussion

This PMS subgroup analysis demonstrated the real-world safety and effectiveness of Isa-Pd in frail Japanese individuals with RRMM, consistent with results from the overall population of Japanese participants in the PMS [9].

While the incidence of infusion-related reactions was underestimated in this study, infectious diseases and infusion-related reactions were more common in frail participants than in those who were grouped as fit/intermediate. Bone-marrow suppression was also more common in frail participants than in fit/intermediate participants; when neutropenia AEs were harmonized according to the PTs for neutropenia, the impact of neutropenia was highest in the frail group. It is possible that the lower incidence of neutropenia across all the subgroups (when compared with the 96% incidence in the ICARIA trial of ISA-Pd [6]) could have been accounted for by the high proportion of patients who had missing frailty scores. Rates of anemia, decreased platelet count, and decreased white blood cell counts were similar in frail and fit/intermediate participants.

The effectiveness of Isa-Pd was comparable between frail and fit/intermediate individuals with RRMM, with no significant differences in ORR and ≥ VGPR between subgroups. Since numerically higher ORRs are indicative of a higher likelihood that the sample is representative of the target population, the low ORR in the frail group should be interpreted with caution. Discontinuations due to disease progression were also similar between groups.

The simplified frailty score used in the current study was originally devised to make use of available real-world clinical information [4, 5]. The original IMWG-devised frailty score used the patient-completed Activities of Daily Living questionnaires to determine the functional status of individuals [3]; however, it was felt that this was not commonly administered to participants and was time consuming. Thus, the simplified frailty score was devised to use commonly available information (i.e., age, ECOG PS, and mCCI) [12]. This simplified frailty classification has recently been externally validated [12] and applied in several studies of individuals with MM [4, 11, 13, 14].

In the RRMM setting, and in line with current results, Isa-Pd had similar efficacy in frail and fitter individuals, when frailty was defined using the simplified frailty score [15]. While there was a trend towards poorer tolerability in frail participants, the overall tolerability profile was manageable in both fit/intermediate and frail participants. The United Kingdom-based retrospective analysis of 106 participants with RRMM treated with Isa-Pd in clinical practice included 72 (67.9%) frail individuals. Median progression-free survival was similar in frail and fit/intermediate participants (10.1 vs 13.7 months, respectively; *p* = 0.5259). Median duration of response (10.1 vs 10.2 months; *p* = 0.685) and median overall survival (15 months vs not reached; *p* = 0.3571) were also non-significantly different between these subgroups, indicating that Isa-Pd was similarly effective in frail individuals. While the incidence of any grade AEs, any grade hematological AEs, and grade ≥ 3 infections were not significantly different between subgroups, grade ≥ 3 hematologic AEs were more frequent in frail than in fit/intermediate participants (58.3% vs 38.2%, respectively; *p* = 0.053) [15].

Although triple regimen therapies are optimal for disease control, it has been suggested that doublet regimens should be the preferred option for frail individuals with RRMM, due to toxicity concerns [16]. However, the current results strongly advocate the benefit of the Isa-Pd triplet regimen in frail individuals in the later-line setting, with clinically meaningful clinical responses (ORR 38.5%; ≥ VGPR 18.0%) and manageable tolerability. While incidence rates of infectious diseases, infusion-related reactions, and bone-marrow suppression were numerically higher in frail participants than fit/intermediate participants, rates of anemia, decreased platelet count, and decreased white blood cell counts were similar in frail and fit/intermediate participants. In addition, rates of discontinuation due to AEs were similar in frail and fit/intermediate participants (10.0% vs 6.9%). Furthermore, clinical response, long-term treatment benefit, and safety in frail individuals treated with Isa-Pd have been previously shown to be consistent with the elderly and the overall general RRMM population, thereby demonstrating the feasibility of this triplet regimen in the frail population [5, 15, 17].

There are limitations to this subgroup analysis. Of note, we were unable to calculate a frailty score for a large proportion of the participants due to a lack of medical history (missing frailty scores), resulting in relatively small sample numbers in the frail and fit/intermediate subgroups. Also, this was not a prespecified subgroup analysis. We recommend that future analyses of the frail population should be specified and carried out in larger sample groups.

In conclusion, although this PMS subgroup analysis was limited by small sample sizes, Isa-Pd was shown to be valuable and well tolerated in frail individuals, making it a potentially useful treatment option for this RRMM population. Close monitoring and dose adjustments may be required in order to manage toxicities and to maintain individuals on the triple therapy Isa-Pd regimen.

## Supplementary Information

Below is the link to the electronic supplementary material.

Supplementary file1 (DOCX 36 KB)

## Data Availability

Qualified researchers may request access to patient level data and related study documents including the clinical study report, study protocol with any amendments, blank case report form, statistical analysis plan, and dataset specifications. Patient level data will be anonymized, and study documents will be redacted to protect the privacy of our trial participants. Further details on Sanofi’s data sharing criteria, eligible studies, and process for requesting access can be found at: https://www.vivli.org/.
