# Supplementary material for: Isatuximab plus pomalidomide and dexamethasone in frail individuals with relapsed/refractory multiple myeloma in Japan
Source: Int J Hematol. 2024 Dec 27;121(4):476–82. doi: 10.1007/s12185-024-03904-y (PMC11923021; doi:10.1007/s12185-024-03904-y)
Supplement: Supplementary file 1 — Supplementary file1 (DOCX 36 KB) [file 12185_2024_3904_MOESM1_ESM.docx]

# Supplemental data

## Supplemental Table 1

Adverse events leading to death by frailty score (n = 9)

| **Cause of death** | **Frail (n = 40)** | **Fit/intermediate (n = 29)** | **Missing frailty score (n = 51)** |
| --- | --- | --- | --- |
| Infections and infestations | 2 (5.0) | 1 (3.5) | 1 (2.0) |
| Pneumonia | 1 (2.5) | 1 (3.5) | 1 (2.0) |
| COVID-19 | 1 (2.5) | 0 | 0 |
| Nervous system disorder | 1 (2.5) | 0 | 0 |
| Subarachnoid hemorrhage | 1 (2.5) | 0 | 0 |
| Respiratory, thoracic, and mediastinal disorders | 0 | 0 | 1 (2.0) |
| Hemothorax | 0 | 0 | 1 (2.0) |
| General disorders and administration site conditions | 1 (2.5) | 1 (3.5) | 1 (2.0) |
| Disease progression | 1 (2.5) | 1 (3.5) | 1 (2.0) |
